# Interactions of Aromatase and Seladin-1: A Neurosteroidogenic and Gender Perspective

**DOI:** 10.1515/tnsci-2019-0043

**Published:** 2019-11-06

**Authors:** Pelin Kelicen-Ugur, Mehtap Cincioğlu-Palabıyık, Hande Çelik, Hande Karahan

**Affiliations:** 1 Hacettepe University, Faculty of Pharmacy, Department of Pharmacology, Sıhhiye Ankara Turkey; 2 Turkish Medicines and Medical Devices Agency (TITCK), Department of Regulatory Affairs, Division of Pharmacological Assessment, Ankara, Turkey; 3 Stark Neurosciences Research Institute, Indiana University School of Medicine, Indianapolis, IN, USA; 4 Department of Medical and Molecular Genetics, Indiana University School of Medicine, Indianapolis, IN, USA

**Keywords:** Aromatase, Seladin-1, Alzheimer’s disease, stroke, epilepsy

## Abstract

Aromatase and seladin-1 are enzymes that have major roles in estrogen synthesis and are important in both brain physiology and pathology. Aromatase is the key enzyme that catalyzes estrogen biosynthesis from androgen precursors and regulates the brain’s neurosteroidogenic activity. Seladin-1 is the enzyme that catalyzes the last step in the biosynthesis of cholesterol, the precursor of all hormones, from desmosterol. Studies indicated that seladin-1 is a downstream mediator of the neuroprotective activity of estrogen. Recently, we also showed that there is an interaction between aromatase and seladin-1 in the brain. Therefore, the expression of local brain aromatase and seladin-1 is important, as they produce neuroactive steroids in the brain for the protection of neuronal damage. Increasing steroid biosynthesis specifically in the central nervous system (CNS) without affecting peripheral hormone levels may be possible by manipulating brain-specific promoters of steroidogenic enzymes. This review emphasizes that local estrogen, rather than plasma estrogen, may be responsible for estrogens’ protective effects in the brain. Therefore, the roles of aromatase and seladin-1 and their interactions in neurodegenerative events such as Alzheimer’s disease (AD), ischemia/reperfusion injury (stroke), and epilepsy are also discussed in this review.

## Introduction

Neuroactive steroids, or neurosteroids, are produced peripherally by exocrine glands, such as the ovaries and adrenal glands, and can cross the blood-brain barrier to influence neuronal signaling [1, 2]. They play important roles in the neuroendocrine control of brain excitability based on their conversion to different metabolites, such as androstenediol and estradiol (E_2_) [3, 4, 5, 6].

**Figure 1:**
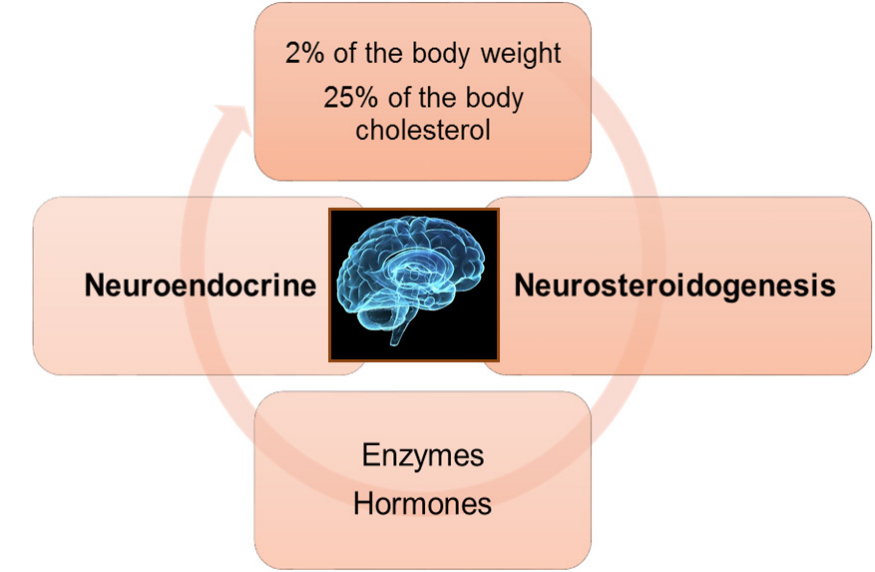
Neuroendocrine and neurosteroidogenic functions of the brain. The brain contains 25% of the total body cholesterol, the main precursor of all hormones [7] and has unique ability of neurosteroidogenesis, which allows the brain to produce specific steroids required for neuroendocrine control and to protect itself from neurodegeneration.

Even though the brain is only 2% of body weight, it contains 25% of the total body cholesterol, the main precursor of all hormones [7]. Brain cholesterol is involved in myelin sheath formation, synaptogenesis, neurotransmission, and neurosteroidogenesis [8, 9, 10, 11, 12, 13]. Although peripherally produced neurosteroids directly influence brain functions, it is well-established that the nervous system is also a steroidogenic tissue and expresses enzymes that are involved in the synthesis and metabolism of steroids. This unique ability of neurosteroidogenesis allows the brain to produce specific steroids required for neuroendocrine control and allows the brain to protect itself from neurodegeneration [14] (Fig. 1).

Two enzymes come into the foreground when discussing neuroprotective activity: Aromatase, which converts estrogen from androgen during the last step of estrogen biosynthesis from cholesterol, and seladin-1, which synthesizes cholesterol from desmosterol (Table 1). The reason why these two enzymes are emphasized in neurodegeneration is hidden in the internal dynamics of the brain. It is well known that estrogens exert neurotrophic and neuroprotective effects by stimulating the expression of neurotrophins and cell-survival factors, enhancing synaptic plasticity, and by acting as antioxidants. Estrogens are protective under numerous types of stressors, including oxidative stress, glutamate excitotoxicity, chemical lesions, traumatic or mechanical injuries, ischemia, iron toxicity, glucose or serum deprivation, and specialized disease related pathogens, such as amyloid beta (Aβ) and HIV proteins [15]. Aromatase-mediated estrogen formation in the brain is known to have regulatory effects on synaptic plasticity, neural stem cell (NSC) proliferation, neurogenesis, newborn neuron migration, differentiation, survival, and neuroprotection [13, 15, 16, 17, 18, 19, 20, 21, 22, 23].

**Table 1:** Comparison of aromatase and seladin-1.

ENZYME	
**Aromatase**	**Seladin-1**
(Estrogen synthase)	(Selective Alzheimer’s Disease Indicator-1; 3-beta-hydroxysterol delta-24-reductase)
**GENE**	
*CYP19A1* Brain specific promoter I.f	*DHCR24*
**LOCALIZATION**	
**Cytoplasmic and presynaptic**	**Endoplasmic reticulum and Golgi**
Over granulosa cells, testis, adipose tissue,	Adrenals,
Placenta,	Pituitary,
Subcutaneous fat tissue,	Thyroids,
Liver,	Ovaries,
Muscle,	Testis,
Normal breast tissue,	Prostate,
Cancer breast tissue,	Liver,
**Brain**	Lungs
	**Brain**
**FUNCTION**	
Androgen → Estrogen	Desmosterol → Cholesterol
Normally synthesized in the nerve cells and regulates neuronal differentiation, neural and synaptic activity and plasticity, neurogenesis, memory and cognitive functions by producing local estrogen.	Normally synthesized in the nerve cells, provides membrane barrier structure and protects neurons from apoptotic cell death by inhibiting caspase-3 activity, Aβ toxicity and oxidative stress.
Its expression increased as an acute response to neurodegenerative damage. Its expression decreased in Alzheimer’s Disease.	Its expression increased as an acute response to neurodegenerative damage. Its expression decreased in Alzheimer’s Disease.

The expression of aromatase is regulated through the alternative use of multiple, promoter-specific first exons (reviewed in [24]). These first exons, which remain untranslated, are spliced into the coding exons 2 through 10 of the aromatase gene, resulting in numerous aromatase transcripts, all of which code for the same protein (reviewed in [24]). Because the brain-tissue specific aromatase promoter (I.f ) is known, designing a drug to increase local estrogen levels in the brain by targeting brain-specific aromatase transcription is theoretically possible [25, 26, 27].

Therefore, increasing steroid biosynthesis specifically in the CNS (central nervous system) without affecting peripheral hormone levels may be possible by manipulating brain-specific promoters of steroidogenic enzymes or by post-translationally modifying their protein isoforms. Regulation of estrogen synthesis in the CNS may be achieved by developing aromatase modulators and can provide new approaches for the prevention and therapy of the neurodegenerative diseases.

## Aromatase and Brain Physiology and Pathology: Estrogen-dependent action

**Figure 2:**
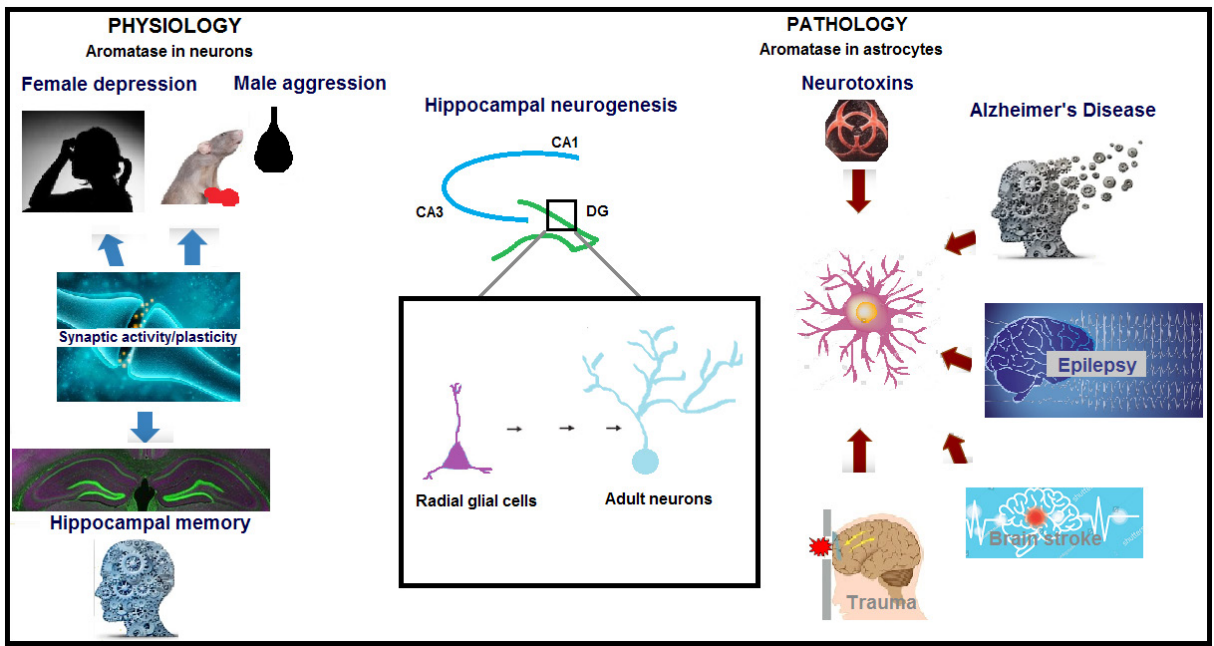
Aromatase in brain physiology and pathology. Brain aromatase is normally synthesized in nerve cells and regulates neuronal differentiation, neural and synaptic activity and plasticity, neurogenesis, memory, and cognitive functions by producing estrogen locally [13, 14, 16, 17, 20, 21, 22, 23]. Aromatase knock-out (ArKO) male mice demonstrated aggressive behavior patterns [30], while depressive symptoms were observed in ArKO female rats [31] and women carrying the *CYP19* polymorphism [32]. The chronic use of aromatase inhibitors in women with breast cancer causes damage to visual and spatial memory [33, 34]. Glial aromatase expression increases in the early stages of neurodegenerative damage and triggers protective local estrogen synthesis [18, 23, 41]. Neurotoxic and mechanical lesions, head trauma, ischemic insults, such as middle cerebral artery occlusion (MCAO), and global brain ischemia result in *de novo* enzyme expression in reactive astrocytes. Aromatase expression and local estrogen production also regulate hippocampal neurogenesis [58, 59, 60].

First, the role of aromatase in physiological events will be discussed before elaborating on its role in brain pathology (Fig. 2). Aromatase is the key enzyme catalyzing estrogen biosynthesis from androgen precursors, such as testosterone. It is mainly a member of the cytochrome P450 enzyme family and is encoded by the *CYP19* gene [28].

Aromatase and its neuroprotective activity in the brain were discovered by Naftolin in 1976 [29]. Brain aromatase is normally synthesized in nerve cells and regulates neuronal differentiation, neural, and synaptic activity and plasticity, neurogenesis, memory, and cognitive functions by producing estrogen locally [13, 14, 16, 17, 20, 21, 22, 23].

When the aromatase gene was examined in detail, researchers discovered that it was encoded by different promoters in different tissues (reviewed in [24]). In vertebrate brains, aromatase is synthesized primarily through promoter I.f in the hypothalamus, hippocampus, and amygdala [27].

Studies conducted to understand the physiological significance of aromatase showed that aromatase knock-out (ArKO) male mice demonstrated aggressive behavior patterns [30], while depressive symptoms were observed in ArKO female rats [31] and women carrying the *CYP19* polymorphism [32]. The chronic use of aromatase inhibitors in women with breast cancer causes damage to visual and spatial memory [33, 34]. Aromatase inhibition in CA1 pyramidal hippocampal neurons or *in vitro* siRNA silencing of aromatase in neuronal cultures results in decreased numbers of dendrites and synapses that cannot be restored by exogenous estrogen therapy. ArKO rats are more susceptible to excitotoxic damage compared to normal rats after chronic systemic or intracerebroventricular aromatase inhibitor administration [35, 36, 37, 38].

While neuronal aromatase predominates in physiological events, astrocytic aromatase participates in pathological conditions [39, 40, 41, 42, 43]. Indeed, glial aromatase expression increases in the early stages of neurodegenerative damage and triggers protective local estrogen synthesis. The neuronal aromatase that is predominant during physiological conditions is replaced by glial aromatase, especially around damaged neurons. While neurons continue to produce aromatase, glial cells begin to produce *de novo* aromatase synthesis around the damage site [18, 23, 41]. Neurotoxic and mechanical lesions, head trauma, ischemic insults, such as middle cerebral artery occlusion (MCAO), and global brain ischemia result in *de novo* enzyme expression in reactive astrocytes. Increased aromatase expression in astrocytes after brain injury is followed by a significant increase in enzymatic activity and increased levels of E_2_ and estrogen receptor-alpha (ERα) in the brain. Therefore, it can be said that aromatase-mediated estrogen conversion from steroid precursors is an endogenous defense mechanism of the brain against neurodegeneration [41, 43, 44, 45, 46].

Adult neurogenesis (Fig. 2) involves aromatase activity and has been studied extensively in zebra finch [47, 48, 49, 50, 51] and zebrafish (teleofish) brains [52, 53, 54, 55, 56, 57]. In mammals, radial glial cells (RGCs) behave as neural stem cells during embryonic development and transform into astrocytes at the perinatal stage [48, 49, 50]. In fish, they persist during adulthood and maintain neural progenitor properties [52, 53, 54, 55, 56, 57]. When a hippocampal lesion is formed, astrocytes express aromatase and produces estrogen, which then acts as a messenger and allows astrocytes to communicate with the RGCs. RGCs are progenitor cells that express aromatase and are located in the subventricular zone around the brain ventricles. Studies observed that RGCs migrate to damaged sites in the brain and accumulate around lesions. Treatment of these cells with an aromatase inhibitor, letrozole, or silencing of the cholesterol carrier steroidogenic acute regulatory protein (StAR) with siRNA has been shown to decrease the number of RGCs and induce apoptotic cell death. This data confirms that aromatase expression and local estrogen production regulate hippocampal neurogenesis [58, 59, 60].

It is well-known that Aβ accumulates in AD brains. In hippocampal neurons, estrogen has been shown to protect neuronal cells from Aβ-mediated cell death by decreasing Aβ production and increasing Aβ clearance (as reviewed in [61]). Additionally, E_2_ reduces Aβ-induced calcium elevation (Ca^2+^) in hippocampal neurons [62] and prevents the hyperphosphorylation of tau, another pathological hallmark of AD (as reviewed in [63]). Importantly, brains of late-stage AD patients have significantly reduced aromatase expression, particularly in vulnerable brain regions that are also deprived of estrogen protection [64, 65]. However, clinical trials using hormone replacement therapy (HRT) and selective estrogen receptor modulators (SERM) to mimic the neuronal protective activity of estrogen in AD patients were unsuccessful and were abandoned due to the side effects. Factors, such as age, basal cognitive activity, genetic background of the patients, disease stage, and duration of treatment were different among patients and likely contributed to the failure of HRT and SERM treatment [23, 63, 64, 65]. When the reasons of this failure were studied at the cellular level, local estrogens and aromatase were also found to be responsible [66]. Studies in hippocampal brain slices have revealed that local concentration of estrogen is six times higher than serum concentration [67]. Neuronal estrogen receptor (ER) expression is known to be mediated by local estrogen synthesis, and neuronal estrogen can only be provided by local aromatase activity. When local aromatase expression and, therefore, local estrogen synthesis is inhibited, neuronal ER expression and the protective effect of estrogen disappear, regardless of how much estrogen is given exogenously [67].

These reports suggest that estrogen can only be neuroprotective if sufficient estrogen levels are present in the brain before morphological changes, such as Aβ plaque formation, occur in AD. Therefore, estrogen treatment could prevent or slow down the development of AD. Once the brain has been deprived of estrogen for an extended time, the protective effect no longer occurs, and subsequent hormone treatment might even be detrimental to cognition [66, 67].

## Seladin-1 and Brain Physiology and Pathology

Studies to investigate the causes of AD have resulted in the discovery of a new protein approximately 20 years ago by Greeve [68] using a differential mRNA display approach. This approach identified a novel gene, named seladin-1, the abbreviation for Selective Alzheimer’s Disease indicator-1, which was differentially expressed in selective vulnerable brain regions of AD patients, such as the hippocampus, amygdala, inferior temporal cortex, and the entorhinal cortex. Down-regulation of seladin-1 expression in these areas was paralleled by increased hyperphosphorylated tau, a protein component of neurofibrillary tangles, and neurodegeneration [68, 69, 70, 71, 72].

A later study showed that this protein is actually a well-known enzyme, 3-beta-hydroxysterole delta-24-reductase, that is encoded by the *DHCR24* gene and was identified as a human homolog of the *DIMINUTO/DWARF1* gene described previously in plants [73]. Seladin-1/DHCR24 is highly conserved and expressed in the CNS, especially in neurons under basal conditions [70]. It catalyzes the last step in the biosynthesis of cholesterol from desmosterol [74]. The deficiency of seladin-1/DHCR24 has been shown to decrease cholesterol levels in the plasma membrane and subsequently, reduce the formation and stability of lipid rafts [75]. Thus, as lipid rafts are important for mediating cell function [76] downstream cellular signaling is also affected [68]. Seladin-1/DHCR24 has also been reported to play a role in cellular responses against oxidative and oncogenic stress and inflammation [77, 78, 79, 80, 81, 82]. Independent from its enzymatic activity, seladin-1 protein has been shown to protect neurons exposed to Aβ and oxidative stress from apoptotic cell death [70] and inhibit caspase-3 activity responsible for apoptosis [83].

Recently, the interactions between seladin-1 and estrogen were investigated. Fetal neuroepithelial cell (FNC) cultures were used to study the relationship between seladin-1 and estrogen. These cells synthesize ERα and β, as well as seladin-1. An increase in seladin-1 mRNA was observed in these cells after treatment with 17β-E2 and the SERM, tamoxifen. Again, a significant increase in seladin-1 expression was detected after FNC treatment with a selective ERα agonist. These investigators also questioned whether there is an estrogen response element (ERE) on the seladin-1 gene and they identified a possible binding site on the seladin-1 promoter. ERα and the seladin-1 promoter was co-transfected to these cells. Treatment of the cells with 17β-E_2_ and SERM resulted in seladin-1 luciferase activity proved that seladin-1 gene has ERE. These studies also indicated that seladin-1 is a downstream mediator of the neuroprotective activity of estrogen [69].

**Figure 3:**
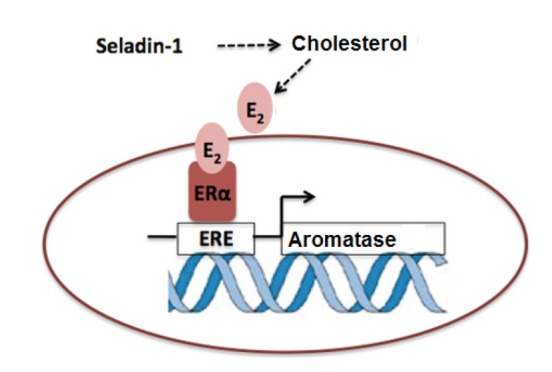
Schematic of the interaction between aromatase and seladin-1. Decrease in brain E_2_ levels is perceived as stress by neurons, and brain aromatase levels increase as a protective/compensatory mechanism [19]. After aromatase inhibition, seladin-1 levels increased as a protective mechanism to compensate for the decline in E_2_ synthesis. Then, E_2_ binds the estrogen response element (ERE) on the aromatase gene and regulates the aromatase promoter for neuroprotective activity of estrogen [69].

In our study, the aromatase inhibitor, letrozole, increased seladin-1 protein levels significantly in human neuroblastoma cells (SH-SY5Y). It is known that a decrease in brain E_2_ levels is perceived as stress by neurons, and that brain aromatase levels increase as a protective/ compensatory mechanism [19]. For this reason, we hypothesized that after aromatase inhibition, seladin-1 levels are also increased as a protective mechanism to compensate for the decline in E_2_ synthesis. To test this hypothesis, we measured the E_2_ level in SH-SY5Y cells and observed that it was significantly decreased in the letrozole-treated group. Thus, increased seladin-1 can trigger an incremental change in cholesterol, the precursor for E_2_ synthesis [84] (Fig. 3).

Seladin-1 is also important in the synthesis of cholesterol, which contributes to cell membrane structure and barrier properties [15, 68, 75, 76]. Studies in rat adrenal gland cells (PC12), SH-SY5Y cells, and FNCs show that seladin-1 protects membrane integrity, which provides resistance to Aβ toxicity [85]. When seladin-1 is silenced, the integrity of the membrane is disturbed, resulting in the formation of Ca^2+^-permeable pores on the membrane and subsequent cytotoxicity due to Ca^2+^ hyperexcitation [85]. Seladin-1 overexpression has been shown to prevent this damage by preserving membrane integrity [15, 68, 75, 76]. When seladin-1 is knocked out, the integrity of the membrane deteriorates, making easier for β-secretases (BACE) to break down amyloid precursor protein (APP) into Aβ, thereby inhibiting plasmin, the enzyme that degrades Abs, and causing Aβ accumulation [15, 68, 75, 76].

In seladin-1 knock-out (SelKO) mouse models, homozygous mice were found to be born with severe dermopathies and die shortly after birth, so experiments are performed using heterozygous mice. AD mice were crossed with SelKO mice (AD/SelKO) in order to mimic the decreased expression of seladin-1 in AD; the membrane and intracellular cholesterol levels were decreased, and desmosterol levels were increased in the AD/SelKO mice. Significant Ab_1-40_ and Aβ _1-42_ increases were also observed in AD/SelKO mice compared to that in the AD mice [75].

In individuals with desmosterolosis, a rare congenital anomaly in humans, there is a significant decrease in seladin-1 enzyme activity due to gene mutations, and serious neurophysiological changes and developmental anomalies are seen [86].

Seladin-1 also plays an important role in nerve protection, similar to aromatase, via its essential enzymatic activity [71, 72]. It is not a coincidence that expression of both aromatase and seladin-1 enzymes, especially in brain regions sensitive to AD, are found to be reduced. Another common feature of these enzymes is an increase in their expression as an acute response to neurodegenerative injury.

## Brain aromatase and seladin-1 expression and interactions in AD

AD, the major cause of dementia, is a progressive neurodegenerative disorder that is characterized by memory loss and cognitive deficits with both genetic and environmental components [23, 70, 87]. Reduced serum estrogen in postmenopausal women has been widely reported to increase the risk of AD and is correlated with AD-related neuropathological changes [88, 89, 90].

The neuroprotective effects of the aromatase product E_2_ are well established in preclinical studies [63, 91]. Although *in vitro* research demonstrated the neurotrophic, neuroprotective [92, 93], neuroregenerative, anti-inflammatory [94, 95], anti-excitotoxic [96], and antioxidant [90, 97, 98] effects of estrogen, the data obtained from clinical studies are conflicting [89, 92, 97, 99, 100, 101, 102, 103, 104, 105, 106] in neurodegenerative diseases, such as AD. Early trials supported the idea that patients with AD could benefit from estrogen therapy by improving deficits in memory and cognitive functions; however, these studies were conducted on perimenopausal women or healthy postmenopausal women [106]. In addition, several trials suggested that estrogen therapy can be effective in preventing AD if the therapy is implemented at earlier ages [99, 100, 101, 102, 103, 104, 105, 106].

It has been shown that estrogen therapy, HRT, could protect at least some postmenopausal women against cognitive impairment, dementia and AD, if the hormone treatment commenced soon after menopause; a later start of HRT may be detrimental [107, 108, 109, 110, 111]. This would concur with the hypothesis of the “healthy cell bias of estrogen action” [112]. Another important factor that could define subgroups of women for potential estrogen therapy is genetic. Polymorphisms related to gonadal steroid synthesis and metabolism could affect the outcomes of HRT. Therefore it is likely that certain variants of the aromatase gene may be associated with the risk of AD in women. It has also been shown that *CYP19* polymorphisms affect the risk of AD in women. Indeed, *CYP19* gene variants could potentially affect the risk for AD by reducing or increasing the conversion of androgens into estrogens, resulting in altered protection against neuronal injury or neurodegeneration through multiple mechanisms [108]. As a result, alterations to plasma estrogen levels cannot always reflect the changes occurring in the CNS, and, despite its protective effect *in vitro*, exogenous estrogen treatment may not have a therapeutic effect [19, 99, 108]. Therefore, the protective effect of local estrogen may be dominant in the brain, rather than plasma estrogen, and the importance of local brain aromatase expression and activity is emphasized as the source of E_2_ in the brain [18, 42, 89, 106, 109].

In humans, sex differences in the development of AD are frequently discussed; for example, the prevalence and severity of AD and the rate of decline is higher and cognitive deterioration is faster and more pronounced in women than in men [110, 111]. In general, the strong decline of circulating E_2_ due to menopause is assumed to be a major risk factor for AD in women [63]. Neuroprotective effects of estrogen, an increased risk of dementia after menopause, and a 19–29% higher prevalence of AD in women compared to men stimulated an investigation into the relationship between estrogen, the estrogen-synthesizing enzyme aromatase, and AD [64, 65, 108, 112, 113].

In 3-month-old female 5XFAD mice, a significant decrease was observed in total aromatase expression, which was accompanied by reduced aromatase protein level in the CA1 and CA3 regions. It is possible that the increased production of Aβ inhibits aromatase expression in the young mice, which, in turn, results in the loss of neuroprotection by E_2_ [91]. This finding indicates that the function of brain-derived aromatase differs between male and female animal models as well.

Furthermore, a transgenic mouse model for AD (APP23 mice), in which the animals develop Aβ plaques and other pathological changes observable in the brains of AD patients [114, 115], was crossbred with aromatase-KO (Ar^−/−^) mice to test the influence of E_2_ on the formation of Aβ plaques. The resulting female progeny, which is Ar^+/−^ and therefore E_2_-haploinsufficient, demonstrated faster and more severe Aβ plaque formation and less effective Aβ clearance than did aromatase-expressing APP23 mice [89]. Ovariectomy of APP23 females, *i.e.*, the elimination of their major source of systemic E_2_, did not mimic the effects of genetically-induced aromatase deficiency that affected all aromatase-expressing tissues, including the brain. These results suggest that brain-derived E_2_, rather than ovary-derived E_2_, counteracts Aβ plaque formation and is, therefore, neuroprotective in female mice. Surprisingly, compared with APP23/Ar^+/+^ mice, Aβ plaque production is reduced in male APP23/Ar^+/−^ mice, suggesting that endogenous testosterone may protect against AD in males and that the neuroprotective role of brain-derived E_2_ may be sex-dependent [116].

**Figure 4:**
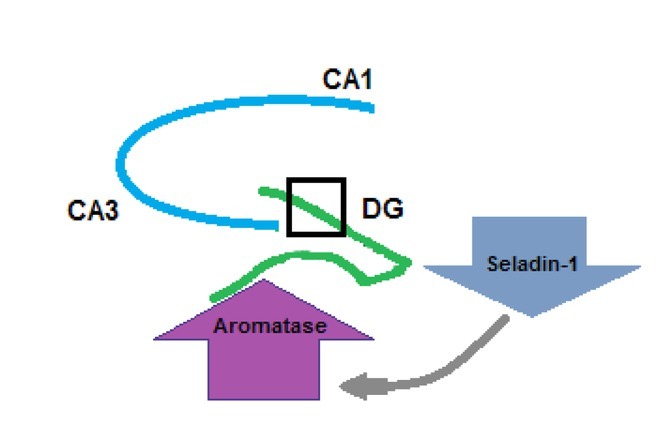
Schematic for the increased aromatase in the DG region of male SelKO/AD mice. Aromatase immuno-reactivity in neurons increased significantly in the dentate gyrus (DG) region of male SelKO/AD mice compared to Sel^+^/AD (control) mice. Thus, we speculated that aromatase levels may increase either to improve neurogenesis in the DG or as a result of neurogenesis after seladin-1 gene expression downregulation in AD

Moreover, in order to investigate interactions between aromatase and seladin-1, we used 5XFAD mice crossed with ArKO or SelKO mice. Using immunohistochemical analysis, we observed that aromatase immunoreactivity in neurons increased significantly in the dentate gyrus (DG) region of male SelKO/ AD mice compared to Sel^+^/AD (control) mice. Furthermore, we also observed that E_2_ levels increased in this group, whereas a significant decrease was observed in the ArKO group. Aromatase increases in the DG of 14–16-week-old SelKO/AD male mice, during the earlier stages of AD [84]. In AD, the aromatase level may increase at earlier stages as a protective mechanism against neurodegeneration. Similar results were observed *in vitro* when serum, the cholesterol source, was removed from cell culture media [42], and *in vivo* as a compensatory response to acute neurotoxin administration [117, 118, 119]. It is tempting to assume that an AD-related increase in aromatase expression, and, therefore, a potential increase in E_2_ synthesis, represents a protective reaction of the tissue to early stages of the developing disease [91]. Furthermore, the alteration of aromatase levels, especially in the DG, can be associated with the role of the DG in neurogenesis [120]. Thus, we speculated that aromatase levels may increase either to improve neurogenesis in the DG or as a result of neurogenesis after seladin-1 gene expression downregulation in AD (Fig. 4). The reason why aromatase increase did not occur in female SelKO/AD mice was not apparent, in contrast to male mice, but may be due to the compensatory effect of higher peripheral estrogen against decreased brain estrogen in these non-ovariectomized young female mice. Brains of SelKO/AD male mice may need more local aromatase and estrogen expression to compensate for less peripheral estrogen, caused by the deprivation of the protective effects of gonadal estrogen relative to females, and the lack of the neuroprotective effects of seladin-1. We suggested that the CNS response to neurodegeneration is reflected as an increase in the local aromatase level. Therefore, the aromatase level does not increase in SelKO/AD female mice because the neuroprotective effect of peripheral estrogen compensates for the changes in local estrogen expression [84]. In women, decreases in gonadal estrogen and local aromatase expression may help to explain their vulnerability to neurodegenerative events and the increase of AD diagnoses after menopause. The sex differences found in the mouse model are in agreement with previous findings, which indicate that the neuroprotective role of brain-derived E_2_ may be more important in females than in males [89, 113, 116], and may help to explain why women are more prone to AD than men [91, 110].

## Aromatase in stroke

Several studies have shown that AD and ischemic brain injury share similar neuropathological features, including altered APP processing, Aβ accumulation [121, 122, 123, 124, 125, 126], and increased neuroinflammation [127]. Cerebral ischemia or “ischemic stroke” is caused by advanced age, hypertension, previous history of stroke or a transient ischemic attack, cardiac arrest, traumatic brain injury, diabetes, cigarette smoking, atrial fibrillation and high cholesterol [128], and results in neuronal death predominantly in brain regions that are most intrinsically vulnerable, such as the CA1 region of the hippocampus [129].

Estrogens display neuroprotective properties and promote neural regeneration after traumatic brain injury and cerebral ischemia by decreasing apoptotic signaling, neuroinflammation, and oxidative stress and by normalizing glutamate concentrations [130]. Aromatase expression increases following brain trauma, suggesting that aromatase plays an important role in neuroprotection by increasing local estrogen levels [23]. These neuroprotective effects of estrogen during brain ischemia have been well-established in ovariectomized rodents and result in a significant decrease in the lesion size and infarct volume [13, 130, 131].

Aromatase plays an important role in endogenous E_2_-mediated protective mechanisms and presents a novel target for neuroprotective therapy in ischemic pathology [19]. Female ArKO mice have significantly increased ischemic damage induced by reversible MCAO in all areas of the examined brain compared to wild-type littermates. The same result was observed when E_2_ production was pharmacologically inhibited by an aromatase inhibitor, fadrozole [19]. Aromatase protein increased after MCAO, and studies evaluating the peri-infarct location and astrocytic localization implicate the potential for aromatase to promote the survival of cells in the penumbra after experimental stroke by local synthesis of estrogens [41]. Peterson et al. (2001) have shown that aromatase mRNA and protein are rapidly and locally upregulated in RGCs, and that these cells migrate to injury sites following neural damage in zebra finch brains. These findings suggest that injury-dependent upregulation of aromatase may be a conserved characteristic of the vertebrate brain and an important component of the initial response of neural tissue to injury [40].

In addition to aromatase expression, transcription of the C/EBPβ protein, a well-known mediator of injury and inflammatory responses in peripheral tissues [132] and potentially, in the brain [133, 134], is increased in ischemic hippocampi and decreased after treatment with the aromatase inhibitor, megestrol acetate. We speculate that, because aromatase is a gene associated with regeneration, it is likely to be a direct transcriptional target of the C/EBP family [135].

Moreover, altered lipid metabolism is also believed to be a key event that contributes to CNS injuries, such as stroke [136, 137]. SREBP-1 is a transcription factor best known for regulating lipid and cholesterol metabolism. The active fragment of SREBP is transported to the nucleus where it binds to the promoters of SREBP target genes, most of which are involved in the synthesis and metabolism of lipids [138]. A recent study reported that, while the detailed mechanisms by which SREBP-1 activation leads to neuronal cell death remain to be established, the researchers discovered a method to inhibit SREBP-1 and, thereby, significantly reduce cell death [139]. In addition, another study showed that the removal of fetal bovine serum, a source of cholesterol, from cell medium results in increased astrocytic aromatase expression and activity [140]. We also observed an increase in aromatase expression when the serum was removed from neuronal cell cultures, and Aβ inhibited this aromatase induction. It is known that Aβ forms lipid micelles in cell culture and, therefore, we speculated that these Aβ-lipid micelles blocked the transfer of SREBP-1 to the active form, produced a false signal that prevented aromatase expression, and blocked the protective increase of aromatase in stress conditions [141]. Moreover, we investigated the effects of indinavir, an inhibitor of nuclear SREBP localization, on the expression of aromatase in rat hippocampi after transient global ischemia. Aromatase and nuclear SREBP-1 protein levels significantly increased after transient global ischemia in the rat hippocampi, and this increase was reduced by indinavir treatment [141]. Taghibiglou et al. (2009) found that SREBP-1 activity increased during oxygen and glucose deprivation, conditions that mimic ischemic stroke, in neuronal cells, and that this activation was blocked by N-methyl-D-aspartate receptor (NMDAR) antagonists. One study also reported that SREBP-1 was activated in a mouse model of ischemic stroke and this activation in affected neurons was an essential step for NMDAR-mediated excitotoxic neuronal death. Inactivation of the SREBP-1 pathway was shown to reduce neuronal damage after stroke in mice [139]. Moreover, another report suggests that the excitatory receptor-dependent activation of SREBP-1 promotes neuronal cell death [142].

Our conclusions from these studies support the central hypothesis that the upregulation of aromatase in ischemic hippocampi and its downregulation in megestrol acetate-treated and indinavir-treated tissues may partly depend on transcription factors, such as C/EBPβ [135] and SREBP-1 [141], respectively. Our findings indicate that ischemia, as well as chronic neurodegenerative processes, lead to increased cytoplasmic aromatase and nuclear C/EBPβ and SREBP-1. Thus, it is possible to hypothesize an interaction between this enzyme and transcription factors [135, 141].

Interestingly, clinical and experimental findings after stroke are abundant and highlight important sex differences [143, 144]. Clinical studies showed that aging women display worse outcomes following an ischemic stroke than men, and that women also have higher mortality after hemorrhagic strokes [145]. In addition, rodents ischemic stroke models demonstrate that young females have smaller infarcted area than young males [146] and that they exhibit less severe stroke consequences during proestrus (high 17β-E_2_ concentration) than during metestrus (low 17β-E_2_ concentration) [147]. During aging, mortality is higher in females than in males, and males display greater bleeding and mortality during hemorrhagic strokes [145]. The study also showed that estrogen treatments improve outcomes in young females and males after ischemic and hemorrhagic strokes, although their effects in aging females during ischemia are controversial [145]. Other clinical and experimental findings also document the impact of sex and sex steroids in other CNS insults [148, 149].

## Seladin-1 in Stroke

A previous study showed that APP and Aβ, or their fragments, aggregate in dense plaque-like deposits in the thalamus of rats subjected to transient MCAO [150]. Subsequently, it was demonstrated that APP processing and the expression of Aβ-degrading enzymes are altered in the ipsilateral thalamus following MCAO, and that this damage remains long after the initial ischemic insult [151]. These alterations coincided with significantly augmented Ca^+2^ levels, depletion of BACE trafficking proteins and increased BACE activity in the ipsilateral thalamus. Based on these findings, the observed alterations resulting in Aβ accumulation could be linked to disrupted Ca^+2^ homeostasis. A non-selective Ca^+2^ channel blocker, bepridil, inhibits BACE-mediated APP cleavage *in vitro* and *in vivo* [152, 153, 154] and significantly decreased Ca^+2^ levels [155]. This decrease was strongly correlated with reduced Aβ_42_ and Aβ_40_ levels in the ipsilateral thalamus of MCAO rats. Seladin-1 decreased at both mRNA and protein levels after MCAO; conversely, bepridil treatment restored seladin-1 expression in the ipsilateral thalamus of MCAO rats and was associated with the improved neuronal survival [156]. The seladin-1 protein is encoded by the *DHCR24* gene and is a potential neuroprotective factor. This notion stems from previous studies, which showed that seladin-1 plays a cytoprotective role in oxidative stress-induced apoptosis by scavenging reactive oxygen species [83]. Moreover, seladin-1 interacts with the p53 tumor suppressor protein [77], a redox-sensitive transcription factor involved in the pathogenesis of brain ischemia and AD. Importantly, seladin-1 expression is downregulated in large pyramidal neurons in specific regions in AD brain and suggests that seladin-1 is associated with selective neuronal vulnerability [68, 69, 70, 71, 72].

From this evidence, it seems reasonable to speculate that neuronal damage accompanied by a reduction in seladin-1, similar to reports in certain AD brains, will have important consequences in the CNS response to ischemia. Thus, paucity in seladin-1 could affect the amount of damage and time of recovery from ischemia, as it is highly dependent on the amount of stress to the affected area [82].

Recently, *Sel+/−* and wild-type (WT) mice were subjected to permanent MCAO. *Sel+/−* mice displayed larger infarct volumes after MCAO than their WT littermates. Moreover, treatment of WT mice with the seladin-1 inhibitor, U18666A, increased ischemic lesions. Inflammation related mediators, such as COX-2, iNOS, TNF-α, and IL-10, were increased after ischemia in *Sel+/−* mice, compared with WT counterparts [82].

## Brain aromatase and seladin-1 in epilepsy

Epilepsy is the most common serious neurological disease and is defined as “a disease of the brain characterized by an enduring predisposition to generate epileptic seizures” [157]. Epilepsy, antiepileptic drugs (AEDs), and the reproductive system exhibit complex mechanisms of action. Reproductive endocrine and sexual dysfunction are more common in patients with partial epilepsy than in those with generalized epilepsy [157, 158, 159, 160, 161, 162, 163, 164, 165, 166, 167], particularly in temporal lobe epilepsy that affects the limbic system, because this area is extensively interconnected with the hypothalamic nuclei that regulate gonadal function [168, 169, 170]. This region controls gonadal hormones, including estrogen, which is converted from testosterone by aromatase. Patients with temporal lobe epilepsy have higher aromatase activity in the cerebral cortex than in subcortical areas. Inhibition of cerebral aromatization results in lower focal availability of estrogen and, theoretically, improves seizure control [171]. While the increase in aromatase expression, and, thus, the synthesis of estrogen, in head trauma, ischemia and AD are neuroprotective, the effects of estrogen on epilepsy are complex. A recent study established that estrogens are epileptogenic [172] and have proconvulsive effects [173]. This effect is due to the ability of estrogens to lower the seizure threshold, thereby increase seizure discharges [172, 174], and to increase neural membrane excitability [173, 175]. However, there have also been some studies that hint at a potential anticonvulsant role of estrogen [175], highlighting not only the complex role of gonadal hormones in the body but also the complex etiology of epilepsy itself. Thus, E_2_ has mixed effects, ranging from no effect, mild anticonvulsant to proconvulsant effects, on seizures that depend on whether physiological or supraphysiological doses have been used [176]. In that sense, aromatase enzyme inhibitors may have great potential for use as an antiepileptic treatment [177]. In some clinical trials, aromatase inhibitors have been used to treat prostate hypertrophy; some success was reported in men with complex partial epilepsy treated using this drug and other aromatase inhibitors [178, 179, 180]. In fact, letrozole, approved for the treatment of breast cancer by the Food and Drug Administration (FDA) [181], has been clinically successful in treating epilepsy in men [178]. In a case study, a 61-year-old man with temporal lobe epilepsy and sexual dysfunction due to low testosterone levels used letrozole to normalize his testosterone level and improve his sexual function and seizure control [178, 180]. Therefore, testosterone supplementation, concomitant aromatase inhibitors [182], and AEDs with aromatase-inhibiting properties should be further investigated as a beneficial treatment for male patients with epilepsy [161, 162, 163, 177, 178, 185, 186, 187].

Therefore, twelve AEDs were tested on commercially available microsomes, from transfected insect cells, for their ability to inhibit aromatase using dibenzylfluorescein as a substrate. The drugs inhibiting aromatase were: lamotrigine, oxcarbazepine, tiagabine, phenobarbital, phenytoin, ethosuximide, and valproate. Gabapentin, primidone, topiramate, and vigabatrin showed no inhibition [177]. Most therapies, apart from inhibiting aromatase, are based on estrogen-lowering drugs and act via various mechanisms, such as linking estrogens to globulins (carbamazepine) [158], binding to estrogen receptors (neurontin and gabapentine–2-[1-(aminomethyl)cyclohexyl] acetic acid), or by inhibiting steroid sulfatase activity (topiramate-sulphamate). These studies suggest that the molecular mechanisms of AEDs are related to either steroid binding or synthesis. Enzyme-inducing AEDs, such as phenobarbital, phenytoin, and carbamazepine, can directly suppress gonadal testosterone synthesis [186], increase hepatic synthesis of sex hormone-binding globulin (SHBG) [187], and increase serum E_2_ levels, either in absolute concentrations or relative to bioavailable testosterone (BAT), and are associated with hyposexuality and hypogonadism [188, 189]. Therefore, a small increase in E_2_ level, presumably as a result of an AED-induced aromatase activity increase, could have a disproportionately large negative feedback effect and contribute to hypogonadism [190].

The effects of AEDs on aromatase and the expression level of its coding gene *CYP19* were examined in the liver, but not in the brain or gonads [191]. Therefore, we investigated the possible effects of the highly efficient, new-generation, antiseizure/anticonvulsant drug, levetiracetam (LEV), on central and gonadal aromatase expression and gonadal tissue functionality. Epileptogenesis was generated in male Wistar rats by an intraperitoneal injection of the excitotoxic agent kainic acid (KA). Significant decrease in the average spike number, amplitude, and frequency by electroencephalography (EEG) were observed in KA-treated rats that received an extrapolated dose (27 mg/kg/day) of LEV from a clinically used human dose (1000 mg/75 kg adult) at all time points when compared to the first day of the experiment. LEV did not affect seladin-1 expression levels in the brains of male rats, suggesting that increases in KA-induced neurosteroidogenic enzyme expression were specific to aromatase. Our study indicates that LEV exerts a slight inhibitory effect on basal central aromatase and attenuates testicular aromatase [185]. These results also correlate with a significant downregulation of *CYP19* in H295R cells exposed to LEV at intermediate (100 μM) and high (175 μM) concentrations *in vitro* [192]. LEV decreases aromatase levels in the testis and increases the seizure threshold, possibly by decreasing systemic E_2_ levels. LEV does not induce testicular aromatase, affect the contractility function of the vas deferens, or induce significant changes to the histology of male gonadal tissues. Therefore, these may partly explain why LEV does not cause reproductive dysfunction. LEV may produce a central alteration in the testosterone/estrogen ratio by decreasing the expression of brain and gonadal aromatase. Furthermore, we suggest that the effects of the antiseizure drugs on central and testicular aromatase may be investigated to determine if they may be beneficial for treating male patients with epilepsy [185].

It has also been suggested that E_2_ enhances neuronal excitability in the hippocampi of male but not female rats [176]. However, contrary to these conclusions, AEDs and epilepsy are often associated with sexual disorders in women as well, such as in hyperandrogenism, menstrual disorders, and ovarian cysts. Patients with epilepsy often show hormonal abnormalities, which may be caused either by epilepsy or by continuous treatment with AEDs [158]. Among women, the relationship between epilepsy and estrogen imbalance is especially striking. For example, it has been shown that approximately 56.5% of women with amenorrhea or anovulatory cycles have EEG abnormalities [172]. Seizure frequency can also be associated with perimenarche, menarche [172, 173], menstruation [172, 173, 174, 193], pregnancy [172, 173], perimenopause [172, 173, 174, 193], and menopause [172, 173, 174, 193]. Catamenial epilepsy, a form of epilepsy in which the periodicity of seizure exacerbation corresponds closely with the menstrual cycle, is thought to affect between 30% to 70% of epileptic females [172, 173, 174, 193]. However, the periodicity of seizures is common among both sexes [174, 177].

The use of aminoglutethimide, a first-generation aromatase inhibitor, has been attempted as an antiepileptic drug in combination with other standard drugs [194]. Letrozole is a third-generation, reversible, nonsteroidal aromatase inhibitor, that was approved by FDA for the treatment of postmenopausal women with hormone receptor-positive or hormone receptor-unknown locally advanced and metastatic breast cancer [195]. A recent preclinical study demonstrated the protective effect of letrozole in preventing kindling induced by pentylenetetrazole (PTZ) in mice [196]. Moreover, letrozole was previously reported to inhibit the testosterone-induced increase in PTZ seizure activity in mice [197]. Letrozole administration prior to KA significantly increased the latency to onset of seizures and reduced seizure occurrence in mice. However, the drug demonstrated no discernible effects on KA-mediated neurotoxicity [6].

It is interesting to note that letrozole is easily transported across the blood–brain barrier after systemic application and exerts an inhibitory influence on hippocampal estrogen synthesis, as it does in other regions of the body in mice [198]. Therefore, it was shown that systemic and local inhibition of aromatase activity by letrozole causes spine synapse loss and significantly impairs (long term potentiation) LTP in the hippocampus of female and ovariectomized mice, but not in male mice. Thus, ovariectomy itself does not influence LTP, and all evidence points to a role of hippocampus-derived estrogen, rather than to a role of estrogen from peripheral sources, for the LTP. In this context, it is useful to consider that testosterone is also neuroprotective in the male brain [199] after letrozole treatment since testosterone is the direct substrate of aromatase [200].

A case report of a 61-year-old woman with long-standing epilepsy, since age 19, with an average seizure frequency of 5–8 per month (93/year; range 83–142/year) and a diagnosis of post-menopausal breast cancer at the age of 56, had halved her seizure frequency to an average of <4 per month. Replacing tamoxifen with exemestane, an aromatase inhibitor, resulted in complete abolition of seizures, and the patient has remained seizure-free for over 4 years. This suggests that aromatase inhibitors may be beneficial as a mono-therapy or an adjuvant for the treatment of epilepsy, and further research in this area is warranted [171]. Computer-based pharmacophore searches using a model based on aromatase inhibition and the enzyme’s structural features can be used to screen for new candidate antiepileptic therapies. In fact, potent aromatase inhibitors and current antiepileptic compounds display significant (over 70%) chemical and structural similarity, and similarity analyses have proposed a number of antiepileptic compounds with a high potential for aromatase inhibition [177].

## Conclusion

The identification of the brain-specific aromatase promoter and the activity of local estrogen through ERα and its downstream regulator, seladin-1, propose these enzymes to be new targets in drug design for the treatment of neurodegenerative diseases, such as AD, stroke, and epilepsy.

Thus, therapeutic approaches that enhance aromatase activity may be targeted to promote neuroprotection in neurodegenerative diseases. Furthermore, systemic delivery of steroidal hormones, such as estrogen, for their neuroprotective effects have some potential risks, such as breast and ovarian cancer. Therapeutic approaches that are alternative to systemic steroid administration will highlight the neuroprotective effects of these steroids and will be advantageous in neuroprotection.

In conclusion, centrally produced gonadal steroids exert numerous actions, including neuroprotection, constitutive expression of estrogen and regeneration.
